# Explainable automated recognition of emotional states from canine facial expressions: the case of positive anticipation and frustration

**DOI:** 10.1038/s41598-022-27079-w

**Published:** 2022-12-30

**Authors:** Tali Boneh-Shitrit, Marcelo Feighelstein, Annika Bremhorst, Shir Amir, Tomer Distelfeld, Yaniv Dassa, Sharon Yaroshetsky, Stefanie Riemer, Ilan Shimshoni, Daniel S. Mills, Anna Zamansky

**Affiliations:** 1 Computer Science Department, Open University, Raanana, Israel; 2 Information Systems Department, University of Haifa, Haifa, Israel; 3 Dogs & Science-Institute for Canine Science and Applied Cynology, Zurich, Switzerland; 4 Computer Science Department, Weizmann Institute, Rehovot, Israel; 5 Faculty of Electrical Engineering, Technion, Israel Institute of Technology, Haifa, Israel; 6 Primrose, Tel Aviv, Israel; 7 University of Bern, Bern, Switzerland; 8 Joseph Banks Laboratories, Department of Life Sciences, University of Lincoln, Lincoln, UK

**Keywords:** Animal behaviour, Computer science

## Abstract

In animal research, automation of affective states recognition has so far mainly addressed pain in a few species. Emotional states remain uncharted territories, especially in dogs, due to the complexity of their facial morphology and expressions. This study contributes to fill this gap in two aspects. First, it is the first to address dog emotional states using a dataset obtained in a controlled experimental setting, including videos from (n = 29) Labrador Retrievers assumed to be in two experimentally induced emotional states: negative (frustration) and positive (anticipation). The dogs’ facial expressions were measured using the Dogs Facial Action Coding System (DogFACS). Two different approaches are compared in relation to our aim: (1) a DogFACS-based approach with a two-step pipeline consisting of (i) a DogFACS variable detector and (ii) a positive/negative state Decision Tree classifier; (2) An approach using deep learning techniques with no intermediate representation. The approaches reach accuracy of above 71% and 89%, respectively, with the deep learning approach performing better. Secondly, this study is also the first to study explainability of AI models in the context of emotion in animals. The DogFACS-based approach provides decision trees, that is a mathematical representation which reflects previous findings by human experts in relation to certain facial expressions (DogFACS variables) being correlates of specific emotional states. The deep learning approach offers a different, visual form of explainability in the form of heatmaps reflecting regions of focus of the network’s attention, which in some cases show focus clearly related to the nature of particular DogFACS variables. These heatmaps may hold the key to novel insights on the sensitivity of the network to nuanced pixel patterns reflecting information invisible to the human eye.

## Introduction

Charles Darwin famously described the use of facial expressions as displays of emotional states in humans and various non-human species (hereinafter referred to as animals) in his seminal work ‘The Expression of the Emotions in Man and Animals’^1^. Nowadays it is widely acknowledged that facial expressions are an important source of information for recognizing emotional states. In humans, facial expressions serve as a primary nonverbal means regulating interactions^2^ and the association between facial expressions and emotional states has long been established by systematic studies in psychology^3,4^. In animals, facial expressions are produced by most mammalian species^5^, and, as in humans, they are assumed to convey information about emotional states^6,7^. Therefore, facial expressions are increasingly studied as potential indicators of subjective states in animal emotion and welfare research.

The gold standard for objectively assessing changes in facial expressions in human emotion research is the Facial Action Coding System—FACS^8,9^. FACS has recently been adapted for different non-human species, including several non-human primates (e.g. orangutans^10^, chimpanzees^11^, macaques^12,13^), marmosets^14^, dogs^15^ and cats^16^. These systems referred to as AnimalFACS are, as in humans, increasingly used for studying animal emotional states (e.g.^17–19^).

A major challenge in identifying standardised facial expressions in dogs concerns the morphological diversity of their heads^20,21^ and overlying dermal structures, such as the inclusion of permanent wrinkles in some breeds. To identify facial emotional expressions in dogs, Caeiro et al.^18^ applied DogFACS to assess the spontaneous response of individuals of different breeds and mixes in naturalistic emotional settings using online videos. Emotions of both positive and negative valence were investigated, including reward anticipation (a positively valenced emotion) and frustration (a negatively valenced emotion), both characterised by expectation of a desired stimulus^16^. Positive anticipation was defined as being induced in situations involving the “[v]isualisation of food or hearing meal/food related word(s); [v]isualisation of leash, hearing walk related word(s)”and frustration was defined as being induced by the “[v]isualisation of a desired resource (toy, food, space) that is or becomes inaccessible”^18^. While Caeiro et al.^18^ found that dogs displayed significantly different facial expressions in distinguishing certain emotional states, there were no distinctive features identified within the context of frustration. Accordingly, Bremhorst et al.^22^ investigated dogs’ facial expressions of positive anticipation and frustration in a controlled experimental setting, unlike that of Caeiro et al.^18^, standardizing also the dog breed (Labrador Retriever). Moreover, the authors used a non-social context to eliminate the risk of interference from previously learned attention getting responses. To experimentally elicit both emotional states studied, a high-value food reward was used as the triggering stimulus in two conditions: the positive condition was predicted to induce positive anticipation (through conditioned food expectation), and the negative condition should induce frustration (i.e. through prevention of access to the expected food reward). Dogs’ facial expressions in these two states were measured using DogFACS. The authors found that the “Ears Adductor” variable was more common in the positive condition, while “Blink” , “Lips Part” , “Jaw Drop” , “Nose Lick” , and “Ears Flattener” variables were more common in the negative condition^22^. In a follow-up study, Bremhorst et al.^19^ tested a new group of dogs using a similar set-up. However, in this study, two different types of rewards were used (food and toys) to test the generalizability of their previous findings to a wider range of contexts^19^.

The previous results were replicated^19^, with four further variables more common in the negative condition: “Ears Downward”, “Lip Corner Puller”, “Tongue Show” and “Upper Lip Raiser”. All of the identified facial expressions except the “Upper Lip Raiser” were independent of the reward type the dogs were expecting to receive^19^. Furthermore, basic measures of diagnostic accuracy were evaluated for the identified facial expressions as potential emotion indicators, including their sensitivity, specificity, and positive and negative predictive values^19^. The results indicated that none of these facial expressions would have provided consistent correct classifications of the associated emotion if used on their own as individual emotion indicators^19^. This does not discount their potential value as signals, but perhaps emphasizes the normal holistic processing of facial configurations^23^, rather than the focus on single elements within it.

The presence of an audience in an emotional context is an important element to be considered when investigating facial expressions (of emotions) in dogs, as shown by a recent study of Pedretti et al.^24^. Similarly to^19,22^, the authors also exposed dogs to positive anticipation, and non-social and non-social frustration, evoking test sessions. They also used DogFACS to analyse dogs’ facial expressions in these situations, apart from other behaviours such as tail wagging, measuring pre and post-test salivary cortisol concentrations. They found that “Ears Forward” occurred more in the positive condition compared to the negative conditions. Furthermore, this variable was positively influenced by the presence of an audience, and negatively correlated to the pre-test cortisol concentrations, suggesting it may be a good indicator of dogs’ level of attention. “Ears Flattener” , “Blink” , “Nose Lick” , “Tail Wagging” and “Whining” (the latter two not included in DogFACS variables) were also associated with the presence of an audience but were not correlated to cortisol concentrations, suggesting a communicative component of these behaviours.

This shows that DogFACS can also serve to investigate dog facial expressions not only as cues (i.e., producing behaviour changes that accompany emotional states) but also as signals (i.e, behaviours specifically produced for the purpose of communicating an emotion to a communication partner), see also^25^. The AnimalFACS systems hence provide an important means of promoting understanding of animal facial expressions. However, the use of these systems for facial expression analysis has its challenges, including its dependence on manual annotation which requires extensive human training and certification, this can be time consuming to undertake, and may be prone to human error or bias^26^.

Automation has the potential to provide an important complementary advancement to this process. In particular it is argued that automated tools to have greater objectivity and reliability than manual coding, eliminating subjectivity and bias^27,28^, but they also do not depend on single feature detection for their success. It is therefore not surprising that automated facial expression coding is a vibrant field in human emotion research, with numerous commercial software tools available, such as FaceReader by Noldus^29^, Affdex^30^, EmoVu^31^, as well as extensive databases such as CAS(ME)$^3$^32^.

In animals, on the other hand, automation of facial exoressions analysis is under-researched. This is due to several challenges (as discussed by^33,34^), including: first the relative recency of growth or interest in animal emotion research, which means much less data are available compared to the vast amounts of data in the human domain. Second, especially in domesticated species, the great variation in facial morphology presents technical challenges^35^. Last, the lack of verbal self-report makes it challenging to establish ground truth for the emotional state experienced in animals, whereas in humans, self-reporting is a standard approach for this purpose. Data collection protocols for animals thus require extensive control and regulation, operational definitions of the emotional states studied (see e.g.^18^), or possibly rating by human experts—although this potentially introduces bias and subjective judgement.

Broomé et al.^36^ provided a comprehensive survey of twenty studies presenting state-of-the-art approaches to automated recognition of emotion and pain in animals. The majority of these works focus on the occurrence of pain. Species that have been addressed in this context include rodents^37–39^, sheep^40^, horses^33,41,42^ and cats^43^. All of these works provide a binary classifier for pain/no pain, using machine learning techniques.

Work on more widely automating animal emotion recognition is much more scarce. Two studies in non-human primates focus on related Action Unit/facial expression recognition, without explicitly addressing emotional states^44,45^. Blumrosen et al.^44^ automated recognition of four facial expressions of non-human primates: neutral, lip smacking, chewing, and random mouth opening with minimal annotation efforts, while Morozov et al.^45^ implemented a prototype system for automatic MaqFACS coding for Rhesus macaques, trained to classify six MacFACS variables.

Only three works providing end-to-end classification for different emotional states were surveyed in Broomé et al.^36^. Corujo et al.^46^ defined four emotional states for horses: “alarmed” , “annoyed” , “curious” , and “relaxed” , defining each of them in terms of eyes, ears, nose and neck behavior. For instance, “relaxed” was defined as eyes: partially to mostly shut, ears: relaxed, opening pointing to the sides, nose: relaxed mouth and neck: approximately parallel. A convolutional neural network (CNN) model was trained to predict these four “classes” of emotion. Ferres et al.^47^ used automated pose estimation using DeepLabCut^48^ for the classification of four emotion classes “anger”, “fear”, “happiness” and “relaxation” for dogs. Franzoni et al.^49^ also used a CNN model to classify limited attributes related to emotional states: “smile” (related to “joy” ), “growl” (related to “anger” ) and “sleep” (related to a neutral state).

Of the three works related to dogs^47,49,50^ two focused on body for recognizing emotional states^47^ and pain^50^, and one on facial expression of emotion^49^. However, the datasets used in the studies of Ferres et al.^47^ and Franzoni et al.^49^ both contained images collected from the internet and annotated by non-experts, and thus potentially were of low reliability and validity. The work of Zhu^50^ studies pain recognition based on body language, and not facial expressions.

The study presented here is the first to explore automated recognition of dog emotions from facial expressions, using a dataset collected from a carefully designed experimental protocol where the context defines the emotional states^22^. In this protocol, the emotional states of positive anticipation (a positive emotion) and frustration (a negative emotion) were operationally defined (in accordance to^18^ and experimentally induced in a sample of 29 Labrador Retriever subjects, minimizing variability of morphological differences between dogs. The facial expressions that the dogs produced were coded objectively using the standardised DogFACS system by certified DogFACS coders. This dataset creates a unique experimental environment for exploring different approaches to automation of emotion recognition with minimal bias in the definition of emotion. The data further benefits from reduced morphological variation of participants’ faces due to the standardisation of the breed.

According to^36^, there are two standard routes to classification of emotional or pain state: using hand-crafted features, or using a deep learning paradigm based on learnt features^51^. Hand-crafted features can be roughly divided into *low level* features, which are based on image statistics (such as histograms of oriented gradients) commonly used in the computer vision literature^51^, and *high-level* features, which are semantically grounded, in species-specific anatomical facial and/or body structure, grimace scales, action units, etc. Examples of the latter are cat facial landmarks^52^, dog body keypoints^47^ or sheep pain action units^40^. These features promote explainability of the machine learning algorithms by grounding the model’s decisions in behavioural concepts. The deep learning approach, on the other hand, is more flexible and expected to perform better (especially when large datasets are available), yet requires costly computational resources and is ‘black-box’ in the sense that it does not lend itself to explaining in human-comprehensible terms why a particular classification decision is made.

In this study, we investigate both of these alternative routes to automated classification of emotional states in dogs. The first route uses DogFACS variables as explainable high-level features. The classification pipeline has two stages in this case: first, automated recognition of DogFACS codes and second, using the annotations to classify the emotions studied. We demonstrate the utility of such explainable representation for understanding the way in which the DogFACS variables are used in the machine’s decision making. The second route takes a (simpler, one-staged) deep learning approach, letting the machine learn directly from the data features that are not necessarily human-understandable. We further compare aspects of explainability between the two approaches, and use heatmap visualization techniques to highlight the relationship of the learnt features to semantic objects related to the dog facial parts.

## Results

### Dataset

**Figure. 1:**
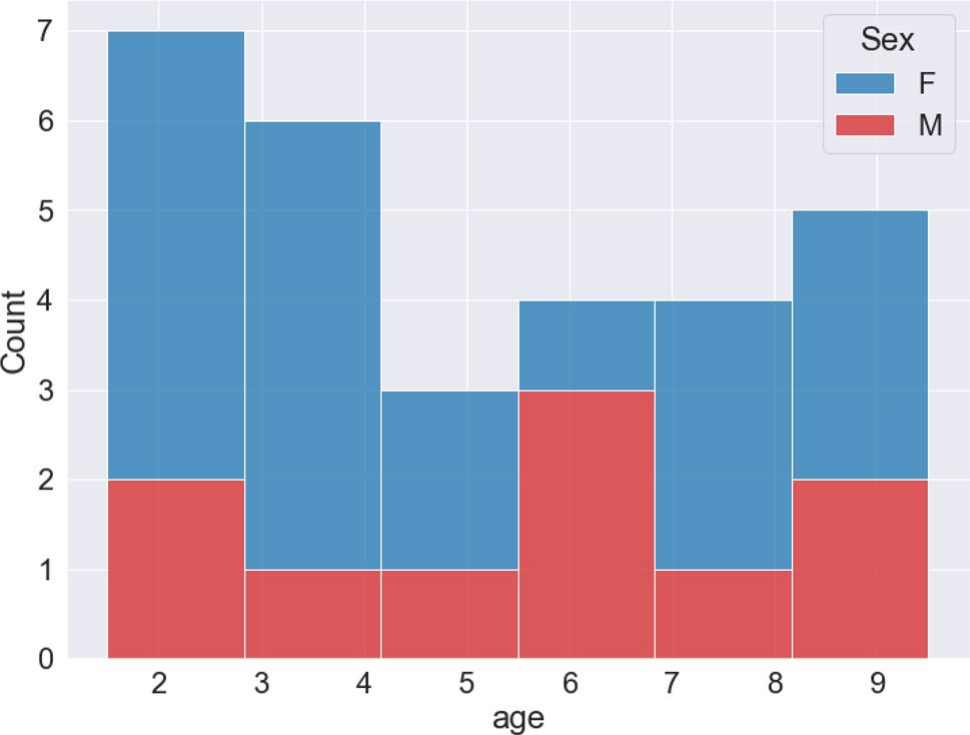
Number of dogs by age and by sex. The dataset contains slightly more female than male dogs, and slightly more younger dogs than older ones.

We used the dataset and DogFACS annotations generated as part of a previous study by Bremhorst et al.^22^. To reduce effects of morphological variation, 29 subjects of one breed without extreme facial features (Labrador Retriever) were tested (19 females–13 neutered, 10 males–9 neutered; age range: 2–9.5 years, mean age = 5.22 years). Figure 1 demonstrates the distribution of the subjects’ age and sex.

**Figure. 2:**
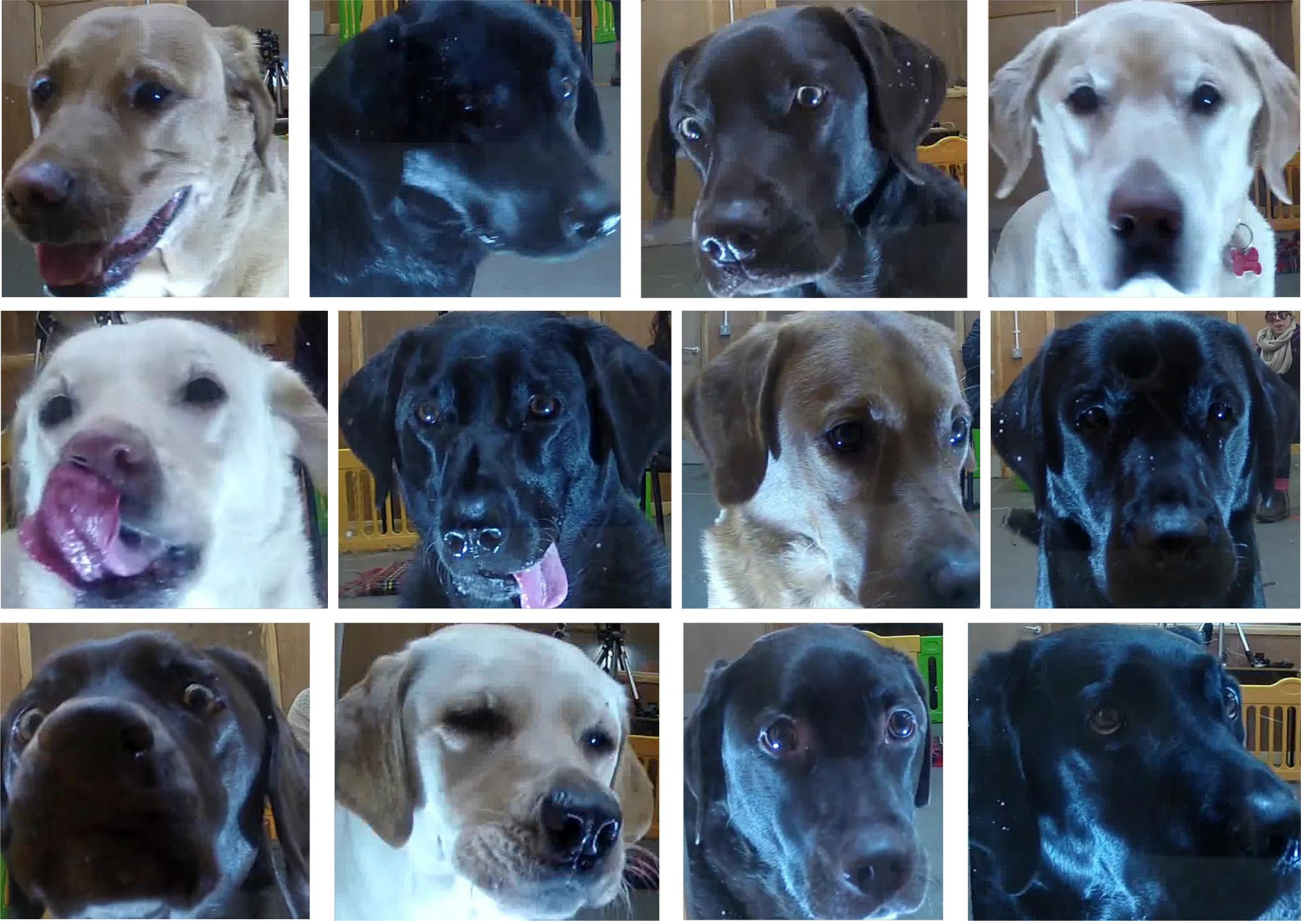
Example frames from the dataset.

The dataset included overall 248 video samples of 3s length recorded in frame rate of 25.25 frames/s, each frame resolution is 1920 × 1080 pixels. The camera used for recording was HIKVision, IR Mini Bullet Network Camera; recorder: HIKVision, DS-7600 Series. The subjects were located behind a transparent window using the protocol which is fully described in Bremhorst et al.^22^. Each subject was tested 3 times in the positive, and 6 times in the negative condition.Thus overall two thirds of the videos were annotated as negative, and one third as positive. It is assumed throughout this study that the negative condition induces frustration, and the positive condition induces positive anticipation, thus henceforth we use the positive/negative valence to refer to the two emotional states. Figure 2 shows crops of dog faces extracted from the dataset.

The dataset was balanced using random undesampling, leaving 82 videos of positive condition, and 82 videos of negative condition from (n = 29) individuals, overall 164 videos. The balancing was done maintaining the same number of positive and negative samples per individual.

All video samples were coded using 39 DogFACS variables based on the DogFACS manual^53^ by a certified DogFACS coder, by annotating one frame per 200 ms using the Solomon Coder (version 15.03.15, Andràs Péter). Out of these 39 variables, eleven variables presented in Table 1 were used in the study of Bremhorst^22^, based on a prevalence of at least 10% across all samples of either the positive or negative condition and at least a substantial strength of intercoder agreement (see^22^ for further details).

**Table 1:** DogFACS variables (Action Units (AUs), Action Descriptors (ADs) and Ear Action Descriptors (EADs)) used in^22^.

Num	DogFACS variable	Description
AU101	Inner brow raiser	Protuberance above the eye moves dorsally and obliquely towards the midline
AU145	Blink	Both eyelids move towards and touch each other, covering the eye for less than 0.5 s
AU12	Lip corner puller	Lip corners move caudally
AU116	Lower lip depressor	Lower lip moves ventrally
AU25	Lips part	Any lip separation
AU26	Jaw drop	Lower jaw moves ventrally in a relaxed manner and teeth are separated
AD19	Tongue show	Tongue is protruded at least until the inner lower lip
AD137	Nose lick	Tongue moves out of the mouth towards the nose and wipes it
AD126	Panting	Mouth is open, tongue is protruded, and dog breathes shortly and quickly
EAD102	Ears adductor	Ears move dorsally towards the midline of the head; bases of both ears come closer together
EAD103	Ears flattener	Ears move caudally

### Overview of the two approaches

**Figure. 3:**
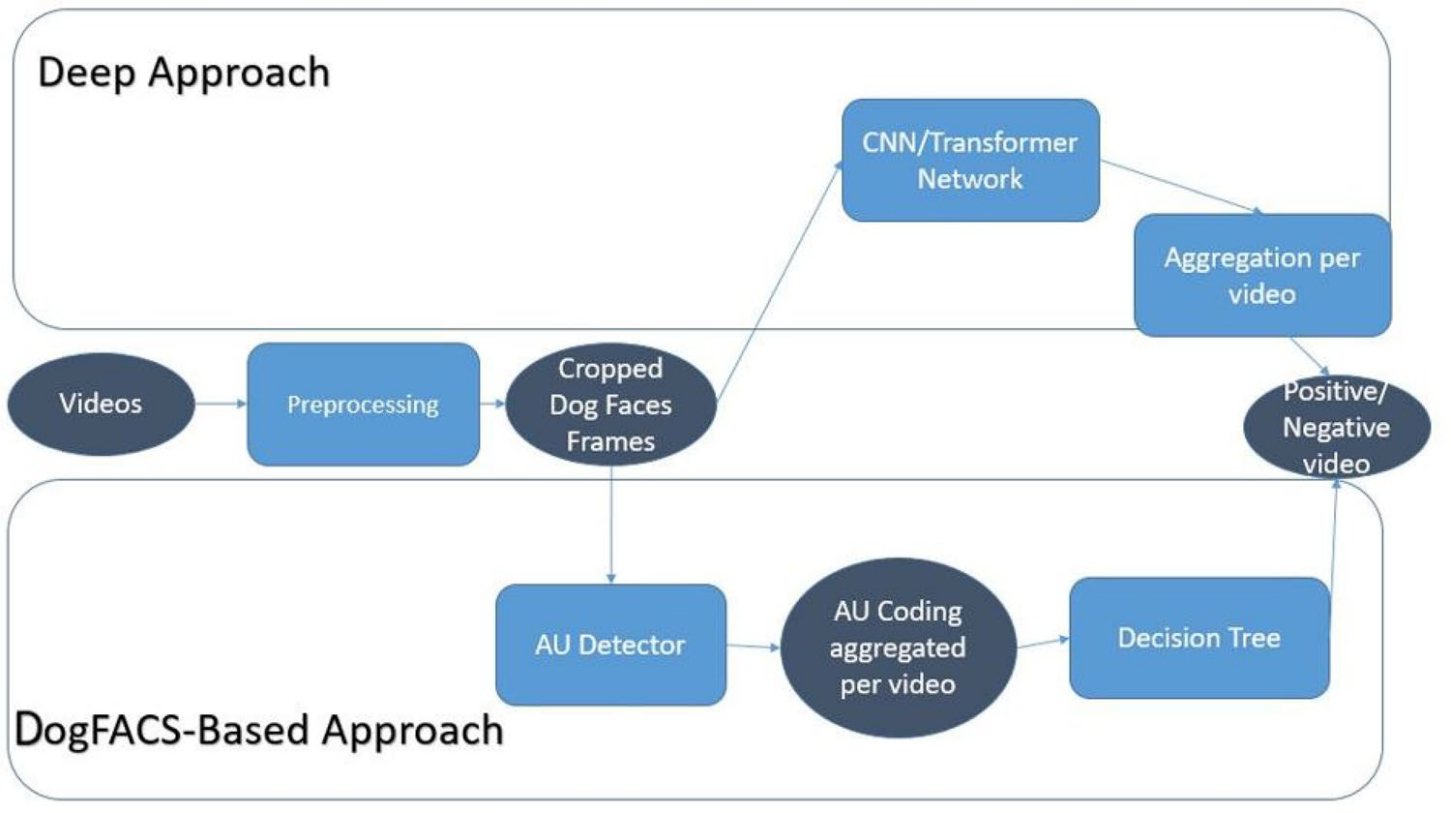
Overview of the two approaches.

We present here a comparison of two different approaches for automated classification of positive and negative conditions: DogFACS-based vs. pure (the DogFACS approach also has a deep learning module for DogFACS variable detection) deep learning approach. Figure 3 presents a high-level overview of the two approaches.

The availability of video data enables us to work with two types of input: single frames, or sequences of frames. The former implies more information loss, but is simpler and more controllable; while the latter includes a temporal dimension, which has been shown to have importance for such tasks, e.g., in the context of detection of pain in horses^42,54^. The prevalent approach in the context of automated recognition of affective states and pain in animals, is, however, the single frame basis (e.g.,^33,39,41,55^). Due to the exploratory nature of this study, we decided on this option.

Thus both approaches work on a single frame basis, i.e., the classification is performed on single frames extracted from videos. However, aggregation of the single-frame information is performed differently in the two cases. After a pre-processing step of extracting cropped dogs faces from the frames (see Fig. 2 for examples), in the deep approach the raw cropped faces are taken as input by a neural network. We experiment here with neural network architectures of two types: convolutional neural network (Resnet50^56^) and the recently introduced vision transformer^57^ (ViT) network. The decisions of the chosen network are then aggregated using majority voting, and the classification decision per video is reached.

The DogFACS-based approach, on the other hand, uses a pipeline with two consecutive steps. The first is the automated DogFACS Variable detector, which detects a set of DogFACS variables in each frame. The DogFACS variables are then aggregated for the whole video. The second step is a decision tree, whose input is the set of DogFACS variables detected in the video is applied to reach the final classification decision.

Thus, the DogFACS-based approach makes a classification decision based on the set of DogFACS variables identified in the video; the deep learning approach, on the other hand, makes a decision on each frame separately, extracting learnt features from raw images, and then aggregates the decision for all frames for the video. Therefore, when exploring explainability of the two approaches, in the former we are expected to have ‘explanations’ along the lines of Bremhorst et al.^22^ (identifying prevalent variables in each of the conditions, or some combination of them). The latter approach, however, is expected to yield more visual explanations on what image features the model focuses, as elaborated below.

For evaluating the performance of our models, we used the standard metrics of accuracy, precision, and recall, which is the standard method in the context of machine learning. As a validation method, we used the leave-one-subject-out cross validation with no subject overlap, which means utilizing each individual dog subject as a separate test set. This method is recommended for datasets in which one individual has more than one associated sample^36^. See Broomé et al.^36^ for a discussion of the importance of choosing an appropriate validation method.

### DogFACS-based approach

#### Sets of DogFACS variables

We experimented with two different sets of DogFACS variables:

1. The set of the eleven variables presented in Table 1 which were utilized in the study of Bremhorst et al.^22^, which are the most promising or potentially most important variables (based on a prevalence of at least 10% across all samples of either the positive or negative condition) and they could be coded reliably (with at least a substantial strength of intercoder agreement, see^22^).
2. The whole set of the 39 DogFACS variables coded in the study of Bremhorst et al.^22^.

#### Classification results

To explore optimal performance, we used the manual DogFACS annotations from Bremhorst et al.^22^ to experiment with different machine learning techniques, including Decision Tree, XGBoost and Random Forest. Table 2 presents a comparison in their performance, with Random Forest performing slightly better for the full set of DogFACS variables (39 variables), reaching accuracy > 71%. In the limited set (11 DogFACS variables), the three models converged to one tree, and thus are presented together, reaching a slightly lower accuracy of > 66%.

**Table 2:** Classifier performance comparison.

Model	DogFACS variables num	Test	Train	Positive (test)			Negative		
		Accuracy		Precision	Recall	F1	Precision	Recall	F1
Decision tree	39	0.71	0.71	0.68	0.69	0.68	0.70	0.73	0.71
XGBoost		0.71	0.71	0.68	0.69	0.68	0.70	0.73	0.71
Random forest		0.72	0.70	0.69	0.68	0.68	0.73	0.75	0.74
Decision tree/ XGBoost/ Random forest	11	0.66	0.66	0.62	0.68	0.65	0.67	0.67	0.67

#### Minimizing the decision tree

**Figure. 4:**
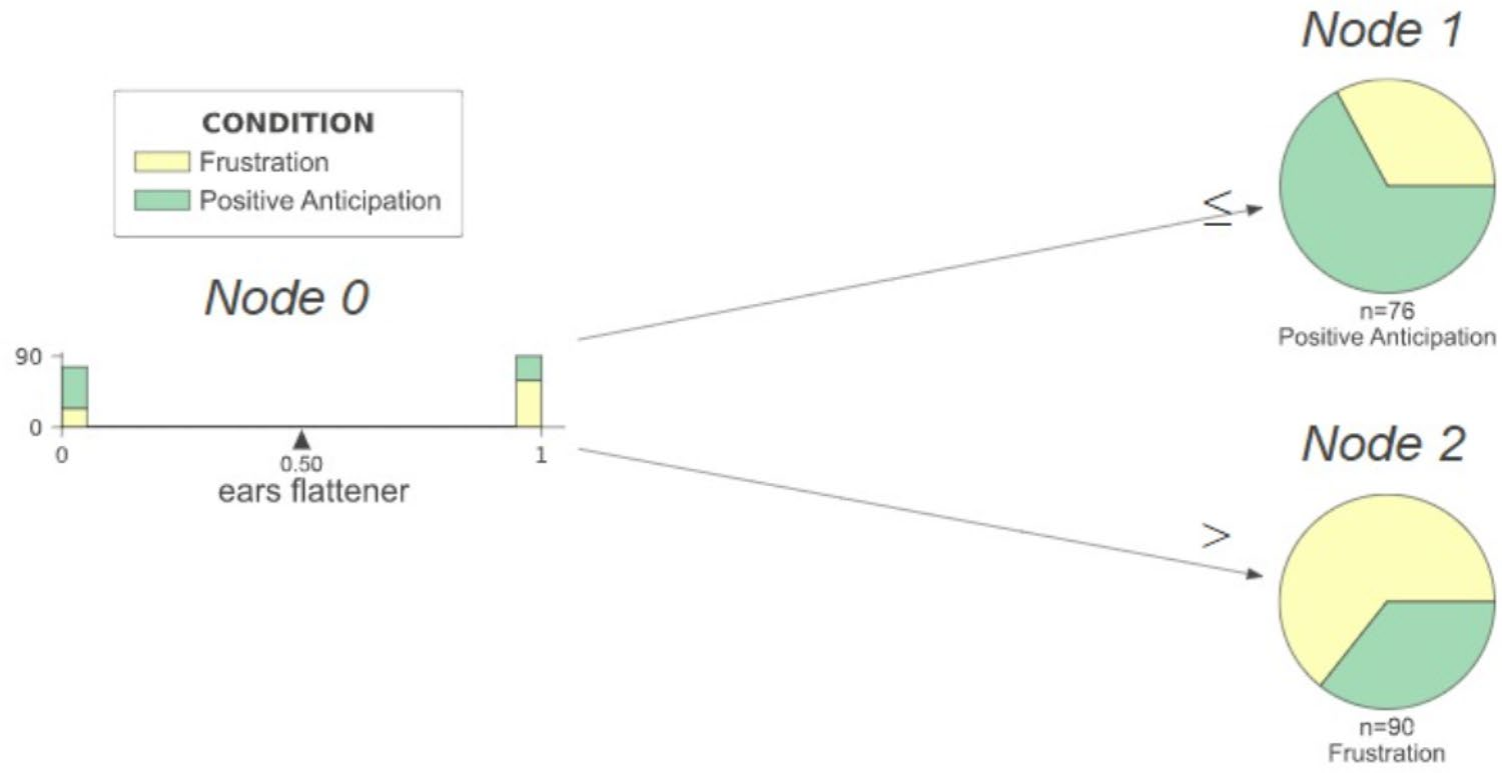
Reduced decision tree (with 11 DogFACS variables).

Next we performed a systematic search for a minimal set of DogFACS variables that would yield the same classification performance presented in Table 2. Table 3 shows that using only one DogFACS variable as a feature guarantees similar performance as the one presented in Table 2. The variable ‘Ears Flattener’ is the most important for classification using the limited set of 11 DogFACS variables, its presence predicting the negative condition. Figure 4 shows the simplified decision tree with just one feature predicting the positive condition—‘Ears Flattener’ absence, and the negative condition—its presence (with accuracy of > 66%).

**Table 3:** Single DogFACS variable predictive performance.

Model	DogFACS variables	Test	Train	Positive (test)			Negative		
		Accuracy		Precision	Recall	F1	Precision	Recall	F1
Decision tree/ XGBoost/ Random forest	Eyes up (1)	0.71	0.71	0.68	0.69	0.68	0.70	0.73	0.71
Decision tree/ XGBoost/ Random forest	Ears flattener (1)	0.66	0.67	0.66	0.62	0.64	0.64	0.67	0.67

Notably, when considering all of the 39 DogFACS variables, ‘Eyes Up’ is the most important variable for classification using all the 39 variables, its presence predicting positive condition with a high accuracy of > 71%.

#### Automated detection of DogFACS variables

Based on our findings, training a detector for the ‘Ears Flattener’ and ‘Eyes Up’ DogFACS variables suffices for a fully automated classification pipeline. We also explored the detection of other variables, using a pre-trained ResNet50 convolutional neural network on balanced datasets (on varying numbers of images due to the variability in DogFACS variable frequency). The performance of the obtained detectors is presented in Table 4.

**Table 4:** DogFACS variable detector performance.

DogFACS variable	Accuracy	Precision	Recall	F1	Num of samples
Ears flattener	0.73	0.72	0.77	0.74	5772
Eyes up	0.71	0.67	0.71	0.69	4096
Ears adductor	0.51	0.32	0.37	0.34	574
Head turn right	0.72	0.76	0.64	0.69	2772
Head up	0.74	0.78	0.69	0.73	3282
Lips part	0.61	0.59	0.62	0.60	2176
Ears forward	0.61	0.73	0.75	0.74	2860
Head down	0.74	0.76	0.76	0.76	3686
Nose lick	0.58	0.46	0.7	0.56	40

### Deep approach

In this approach we employed the common *“transfer learning”* setup, training a linear probe on top of a *fixed pre-trained backbone* using human annotations. We explore the suitability of different backbones for this task by repeating the experiment with four pre-trained backbones: ResNet and ViT trained either in a supervised manner for image classification^57^ or in a self-supervised manner using DINO^58^.

We trained four different models (on the whole dataset) and tested their performance using frames from the same balanced dataset described above (82 videos of the negative condition, 82 videos of the positive condition from (n = 29) individuals, making 164 videos overall).

Table 5 presents classification results analyzed per video, i.e., we say that a video is classified correctly if the majority of its frames is classified correctly. It can be seen that the model trained with a DINO-ViT backbone shows the best performance of above 89% accuracy. Table 6 presents classification results analyzed by frames. As expected, in this case measures are somewhat decreased compared to the analysis done on frames aggregation resulting in  85% accuracy for the model trained with a DINO-ViT backbone.

**Table 5:** DL classification results: frames are aggregated over each video using majority voting.

Backbone		Accuracy	Positive			Negative		
Initial weights	Model		Precision	Recall	F1	Precision	Recall	F1
Supervised	ResNet50	0.81	0.91	0.70	0.79	0.75	0.93	0.83
	ViT	0.82	0.80	0.87	0.83	0.85	0.77	0.81
DINO	ResNet50	0.81	0.92	0.70	0.79	0.75	0.94	0.83
	ViT	**0.89**	0.94	0.84	0.89	0.85	0.95	0.90

Best value is in bold.

**Table 6:** DL classification results: analysis per frame.

Backbone		Accuracy	Positive			Negative		
Initial weights	Model		Precision	Recall	F1	Precision	Recall	F1
Supervised	ResNet50	0.75	0.83	0.64	0.72	0.70	0.86	0.72
	ViT	0.79	0.77	0.82	0.80	0.80	0.75	0.78
DINO	ResNet50	0.78	0.86	0.69	0.77	0.74	0.88	0.80
	ViT	**0.85**	0.89	0.81	0.84	0.81	0.90	0.86

Best value is in bold.

## Discussion

The present study is the first to explore automated recognition of canine emotional states focusing on diverse facial expressions, whilst using a carefully designed controlled experimental setup for dataset creation and annotation. We present classifiers of two different types: deep learning based and DogFACS-based, both having a performance that is comparable to and in some cases outperforms those presented in previous studies addressing recognition of pain or emotional state from facial expressions, including mice^38,39^ (> 89% and 93% respectively), cats^43^ (> 72%), horses^42,46^ (> 75% and 65% respectively) and sheep^55^ (> 64%).

The DogFACS-based approach described here reached accuracy of > 71% using the full set (n =  39) of DogFACS variables, but a lower accuracy of > 66% when using only the eleven DogFACS variables which were utilized in the study of Bremhorst et al.^22^ ( this accuracy was achieved based on manual DogFACS annotations and is expected to drop even lower in an end-to-end pipeline). Of the full set of 39 DogFACS variables, ‘Eyes Up’ were of considerable importance for classification and including them in the Decision Tree leads to higher accuracy (> 71%). However, when interpreting directional variables such as eye movements and their significance as potential emotion indicators, the experimental set-up of the study in which the data were collected must always be considered. In Bremhorst et al.^22^, the experimenter delivered the food reward with a motion slightly above the dogs’ eyeline. This may have encouraged the dogs to look up (inducing the ‘Eyes Up’ variable) in anticipation of food. We must therefore recognize that this DogFACS variable could possibly be an artifact of the experimental procedure. When selecting variables as part of the development of emotion indicators, it is important to weigh up the risk of a type I error (false positive) versus a type II error (false negative) is almost unavoidable. In working with a reduced set of eleven DogFACS variables, we prioritized the avoidance of false negatives over false positives in order not to prematurely exclude a variable from further investigation. We can expect that erroneously accepted variables will be excluded in subsequent studies if their lack of predictive validity is identified (as discussed in^19^).

As a byproduct of these results, we obtained automated detectors for nine DogFACS variables, of which five performed with an accuracy > 70%, demonstrating the feasibility of accurate automated recognition of DogFACS variables. The main challenge for training detectors for each variable is data availability, i.e., the low frequency of appearance of some DogFACS variables, requiring focused efforts for collecting datasets for specific variables. Moreover, some variables have a temporal dimension and cannot be handled on a single-frame basis (e.g., eye blink or panting). Developing detectors for them requires models which also make use of temporal dynamics, such as the approach of Broomé et al.^42^.

It should further be noted that as our dataset is limited to one breed, an immediate future research need is an assessment of the generalizability of the models to other breeds. If performance drops significantly when transferring the results to other breeds, alternative approaches to the deep approach used here are indicated, e.g., in Feighelstein et al.^43^.

Exploring generalizability of the models presented here is important not only in the context of DogFACS variable detection, but also for emotion classification. The dataset used here is controlled not only for breed, but also is recorded in strictly controlled environmental conditions. Generalizing from controlled environments to naturalistic settings is a notoriously difficult challenge also in human affective computing^60^. Feng et al.^61^ provide a review for the human domain of ways in which transfer learning techniques can overcome challenges related to limited amount of data samples, scarce labels, and environmental variability, promoting robust and generalizable automated systems for emotion recognition. Similar ways can be explored in canine affective computing; the results presented here provide a baseline for further exploration of this direction.

Questions such as ’can machines recognize emotional states of animals?’ are interesting in their own right and have far reaching practical applications for animal welfare. The results of our study provide some indication for a positive answer, at least for the case of positive frustration and anticipation in dogs. However, building AI models that recognize dog emotions has a significant added value in helping us understand *how* machines classify emotions, whether they are sensitive to nuances not visible for the eye of a human expert, and what implications it has for our understanding of animal emotions, and ongoing debates on animal sentience. For this reason, it is crucial and promising to explore *explainability* (what is the rationale behind the machine’s decision?), and *interpretability* (how is the model structure related to making such decision?)^62^. These topics are fundamental in AI, and are addressed by a huge body of research^63–65^, with the majority of efforts focusing on deep learning approaches, whose interpretability is limited by their complex structure^66^. Explainability methods are by their nature domain-specific: providing explanations for automated personality trait recognition in job interviews is different, e.g., from providing clinical justification for medical decisions^62^.

Our study is the first to address explainability aspects of AI models for animal emotion recognition. As we compared two different approaches to classification of emotions, there is added value from the ability to compare also the differences in the aspects of explainability they address. The DogFACS-based approach leads to models in the form of simple Decision Trees, which model human logical reasoning in the form of a combination of Boolean conditions concerning the presence/absence of certain DogFACS variables. The explanatory nature of Decision Trees is especially reflected in their simplified version with just one node, such as the one studied here (with ‘Ears Flattener’). Such trees are closely related to concepts useful for human experts, specifically for emotion indicators studied by Bremhorst et al.^19^. Valid emotion indicators are meant to accurately identify a specific emotional state, being present whenever the emotion is present, and absent otherwise. These characteristics are described by sensitivity and specificity, metrics commonly used for assessing the accuracy of diagnostic tests. Bremhorst et al.^19^ found that none of the DogFACS variables considered in the study could be considered a specific individual indicator for positive anticipation or frustration in dogs. Specifically, ‘Ears Flattener’ was shown to have relatively high sensitivity but low specificity. It is thus not surprising that the model described in our study, which is a Decision Tree with ‘Ears Flattener’ as a single feature, did not achieve high performance. However, the relationship between metrics of emotion indicators as used by Bremhorst et al.^19^, and the metrics used here to evaluate the performance of our model is not straightforward. While the former computes sensitivity, specificity, and positive and negative predictive value for the whole, unbalanced data, the latter evaluates performance in a *prediction task*. This means that data is split into two portions: training, which is used to train the model, and testing for its performance evaluation. In contrast to Bremhorst et al.^19^ we also balanced the data using undersampling. However, the intuitive connection between the two is that if an excellent emotion indicator was found using the former approach, we could expect that a Decision Tree using it as a feature would also reach excellent performance.

**Figure 5:**
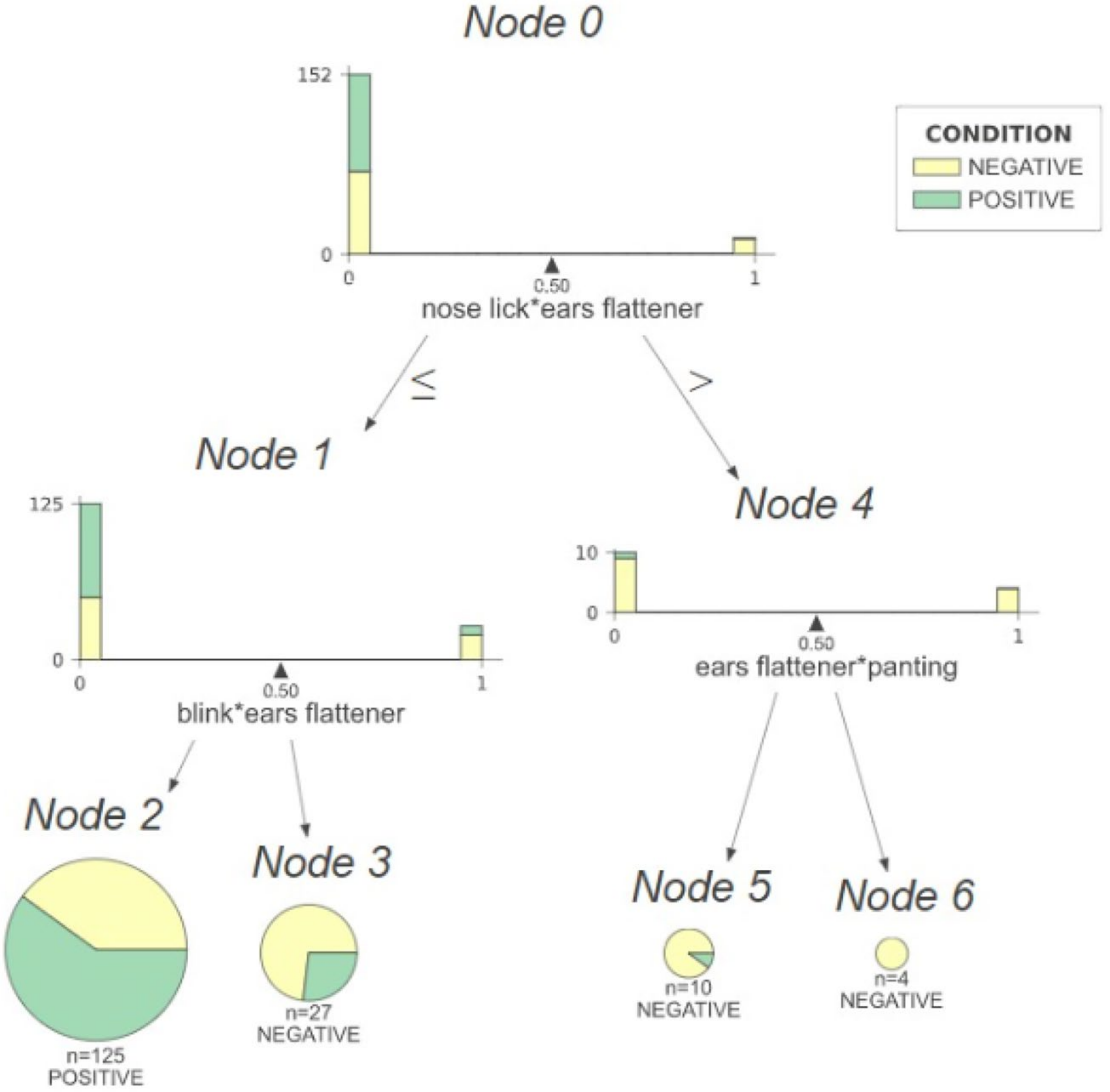
Decision Tree using pairs of DogFACS variables. Accuracy: 0.652463, Precision: 0.651149, Recall: 0.902299.

In addition to explainability, the machine learning approach presented here for searching for optimal Decision Tree models to predict dog emotions has the potential to lead to new insights into emotion indicators. As discussed above, discovery of accurate emotion indicators in terms of Bremhorst et al.^19^ is closely related to the problem of finding Decision Tree classifiers with a single DogFACS variable for emotion prediction. While such classifiers have not been shown to have high accuracy in our study (and indeed, no accurate emotion indicators have been discovered in^19^), classification performance can be improved by considering more sophisticated forms of Decision Trees, for instance by grouping DogFACS variables together into pairs, triples, etc. Our preliminary experiments using pairs of DogFACS variables as nodes, shown on Fig. 5, show that this improved the model’s performance in terms of recall. Importantly, the investigation of which combinations of DogFACS variables may improve classification, can be made in an automated, exhaustive and systematic manner, potentially leading to more fine-grained notions of emotion indicators. This provides a promising path for future research.

The deep learning approach, on the other hand, achieved markedly higher performance of above 89%, demonstrating the potential of such approaches for classification of emotions. Moreover, DINO-ViT backbone seems to be most suitable for the task of emotion classification out of all the four investigated options. We hypothesize that this is due to DINO-ViT features being sensitive to object parts, as shown in^67^; and due to the nature of the emotion classification task, requiring understanding at the object-part level (face parts such as eyes, ears, etc.). Intriguingly, the backbones pre-trained with DINO produce better results than the supervised backbones.

It should be noted that the deep learning classifier worked on the basis of images, then aggregating the results per video. This implies that despite many of the frames do not exhibit the presence of DogFACS variables, the model is still successful in their correct classification. This may indicate the sensitivity of the model to fine-grained details on a pixel level which may go beyond the ability of the human eye. However, it may also be related to potential pitfalls in the form of some inherent bias. Also, the ‘Eyes Up’ variable, discussed above, may have been instrumental for the network and its effect on the decision making is not easily neutralized in the deep learning network. Investigating these issues requires further data collection in different experimental and environmental conditions to rule out such pitfalls.

**Figure 6:**
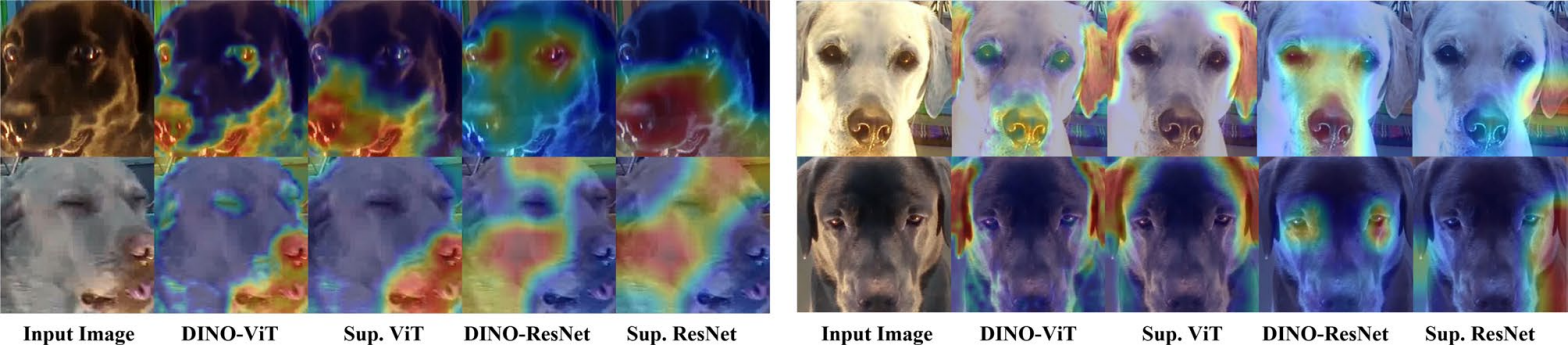
EigenCAM^59^ activation maps on several images for our four different models. The images in the top and bottom row are from positive and negative classes respectively. The DINO-ViT backbone addresses similar areas to those proposed by human annotated DogFACS variables.

Explainability of the deep learning approach considered here is, on the other hand, of a completely different, more visual nature compared to that based on DogFACS. Unlike Decision Tree models, it is extremely challenging to explain decision making of neural networks in human-comprehensible terms, due to their highly complex, ‘black-box’ nature^68^. Using the EigenCAM^59^ method highlights differences between the different models we experimented with (ResNet/ViT, supervised/DINO). As demonstrated in Fig. 6, there are some differences between the models. The ViT models seem to exhibit better localization than the ResNet models, as the highly activated regions (marked by red) are smaller and lay on more salient regions (e.g. eyes, ears, nose rather than skin). Moreover, the DINO-ViT model seems to activate on multiple salient regions rather than one (e.g. activating on the ears, eyes and nose rather than just the ears on the top-right example). We attribute the success of ViT based models to the ability of ViTs to provide a more localized signal than the ResNet models. This stems from their architecture—the resolution of ViT features remains constant throughout the layers, while the resolution of CNN features diminishes as the layers become deeper.

**Figure 7:**
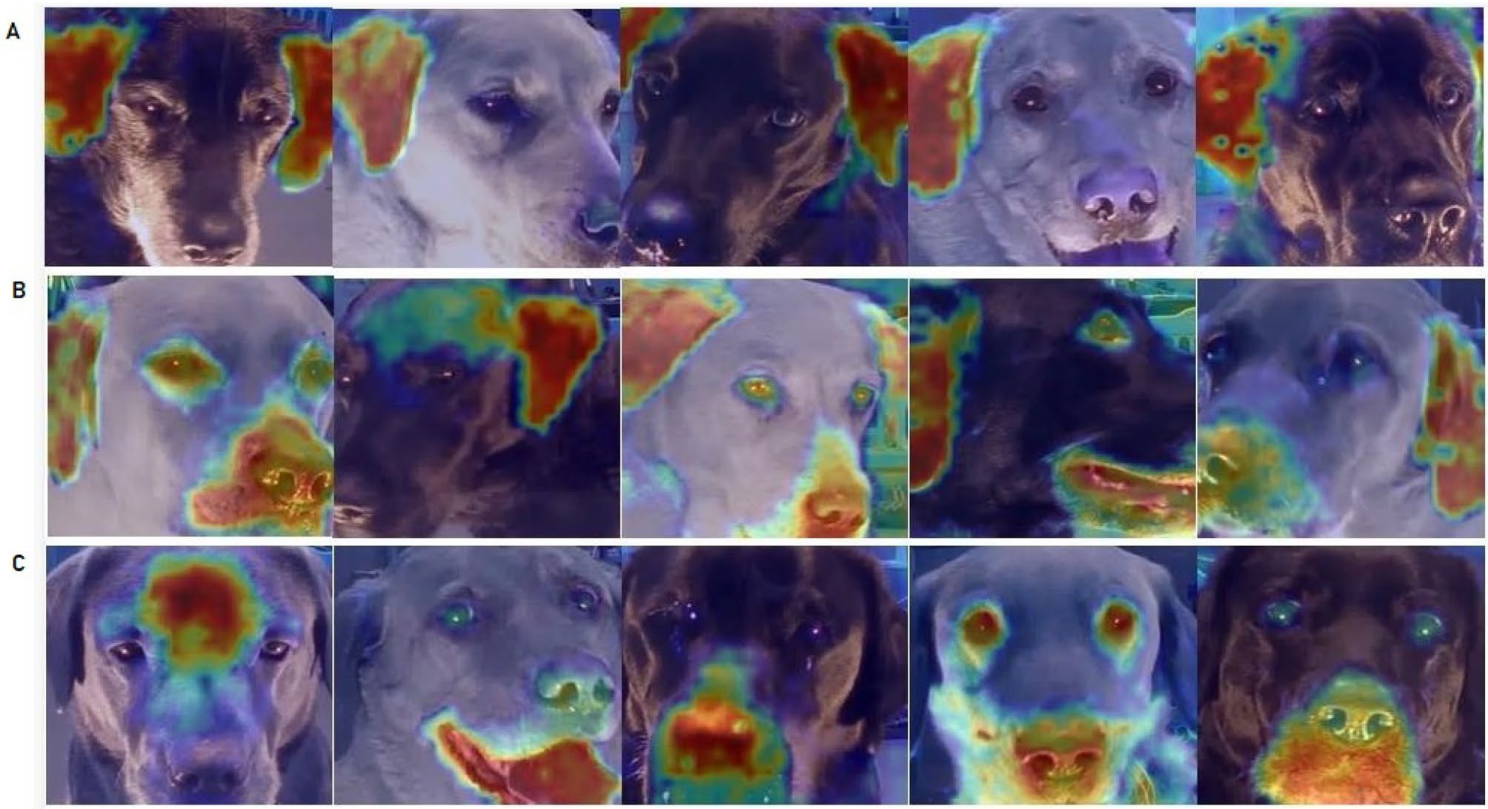
Exploring frames with ‘Ears Flattener’ correctly classified as negative condition. Category (**A**) focus only on ears; Category (**B**) focus on ears and other facial parts; Category (**C**) focus on other facial parts.

**Figure 8:**
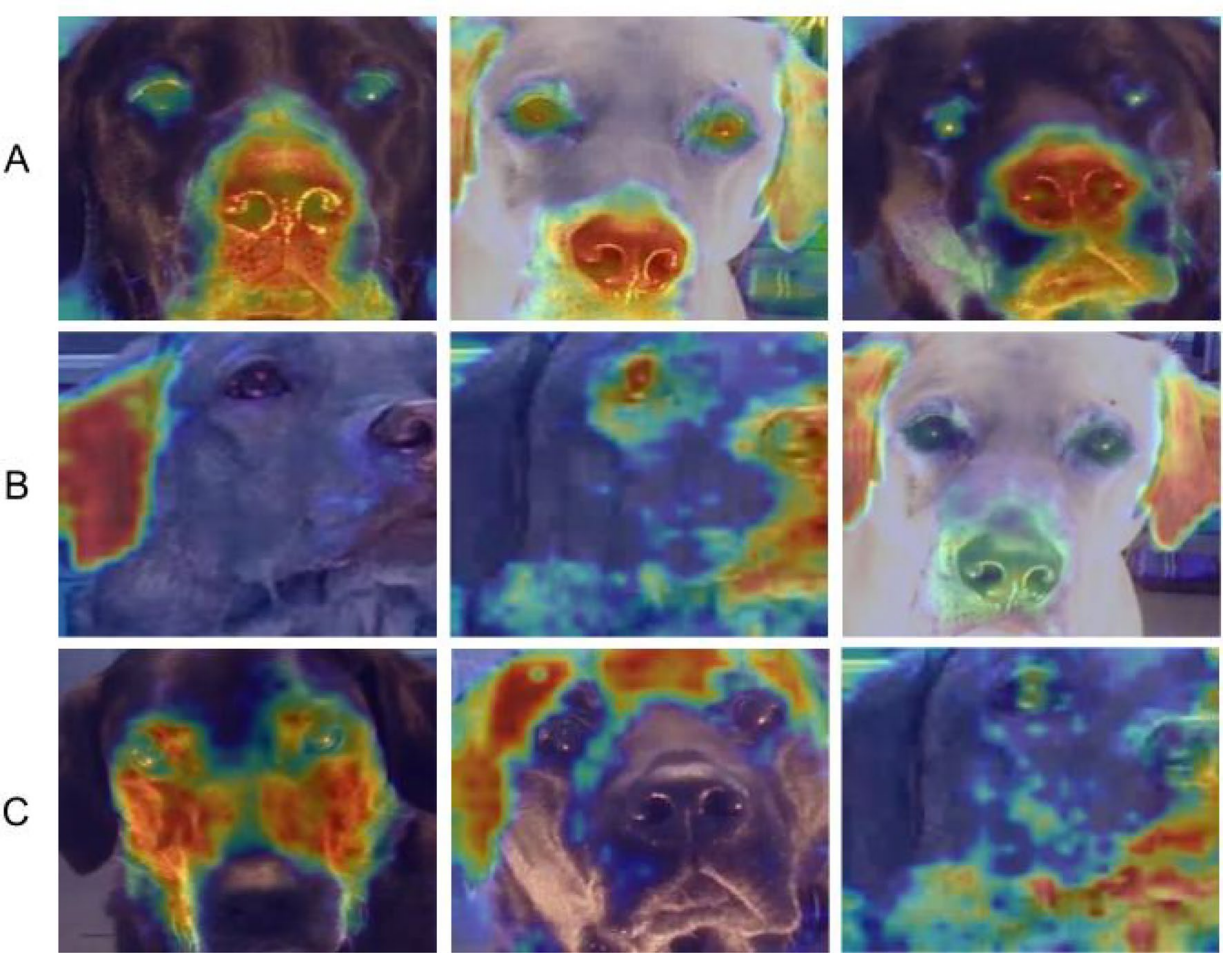
Exploring frames with no DogFACS variables correctly classified by the DNN. Category (**A**) focus mainly on nose-mouth area; Category (**B**) focus on other facial parts; Category (**C**) focus is blurry and not clear.

While reaching definitive conclusions requires further research, we experimented with the EigenCAM method focusing our attention on frames satisfying the following conditions: (i) manually coded with the ‘Ears Flattener’ variable, and (ii) belonging to the class of video samples of the negative condition, and (iii) correctly classified by the DINO-ViT network as negative condition. In our analysis, we divided the examples into three categories, as demonstrated on Fig. 7. Examples of category A are heatmaps with a clear focus on the ears only. This can be seen as consistent with DogFACS-related ‘Ears Flattener’ explanation, i.e., it may be the case that the model learnt patterns related to ear movement. Category B is also consistent with this, showing heatmaps focusing on both ears and other areas, such as eyes, forehead, nose and mouth. The latter may also be indirectly related to the ‘Ears Flattener’ movement, as well as to other DogFACS variables or some other postural feature which may be present in the frame. The most intriguing category, however, is category C: here the model picks up on signals from facial parts other than ears, still making the correct classifications. These cases may hold the key to understanding the sensitivity of the network to nuances not obviously visible to the human eye. In any case, it should be noted that DogFACS annotations cannot exhaustively cover all possible changes in facial behavior, which may be reflected in pixel patterns to which the network is sensitive. We then also extracted heatmaps from videos which had no annotated DogFACS variables. There were nine videos with no variables, eight of them ‘positive’ and one—‘negative’. Strikingly, the majority of these videos (77%) were still classified correctly by the model. This may be another indication of the model picking up on subtle facial behavior not captured by DogFACS. When examining the heatmaps produced for frames of these videos we observed that the nose-mouth area were a main focus for the model. Some other frames show focus on other facial parts, while there are cases of correctly classified frames but blurry and unclear heatmaps. Examples from these three categories are shown on Fig. 8. Interestingly, these heatmaps lack focus on specific facial parts, suggesting that indeed in these cases visual cues were less evident for the model.

Another notable issue related to both approaches with respect to performance is the short length of the videos (3 s) in the current dataset. Using longer videos leads to the challenge of identifying an optimal time window during which an internal state can be considered as constant. This problem has been considered in^69^ in the context of low grade orthopedic pain in horses, and is an important direction for future research also for canine emotional states.

To summarize, this study demonstrated the value of two different automated classification approaches for two emotional states in dogs based on their facial expressions: a positive vs. negative condition. Both of them reached good accuracy comparable to other state-of-the-art methods in automated recognition of animal affect. These results not only provide for the first time an affirmative answer to the question ‘can machines recognize positive/negative dog emotions?’, but also open up new research paths of exploring how machines recognize them, and how to make this recognition explainable to humans. Further experimentation with larger datasets with broader participant characteristics will also promote our understanding of how to develop good animal emotion indicators. One specific direction which seems particularly promising is exploring the potential of approaches related to facial landmark detection, such as OpenFace^70^ and Google MediaPipe^71^. Similar approaches are just beginning to be explored for non-human animals, see, e.g. the study of Feighelstein et al.^43^ on cat faces. Like in the human domain, their development will require extensive multidisciplinary efforts for large dataset collection for various species.

## Methods

### Dataset

The dataset relating to the dogs used for this study was collected previously under the following ethical approvals of the University of Lincoln, (UID: CoSREC252) as per Bremhorst et al.^22^ with an amendment to this research was obtained from the University of Lincoln for using the original dataset in the present study. The current protocol using this data was reviewed by the Ethical Committee of the University of Haifa and no further approval was required.

### Cropping and preprocessing

This step is relevant for both the DogFACS and deep approaches. The original video frames contain background clutter including the surrounding room, humans, dog body, etc. We aim to focus on the *facial expressions* of the dogs and avoid learning other emotional state predictors (e.g. dog body postures). Hence, we trained a Mask-RCNN^72^ to identify canine faces, and used it to crop the facial bounding box from each image. We trained the Mask-RCNN on roughly 200 annotated images from this dataset, making it most suited for this specific experimental setup. Examples of facial crops acquired using the pre-processing stage can be seen in Fig. 2.

### DogFacs-based approach

#### From videos to DogFACS variables

**Figure 9:**
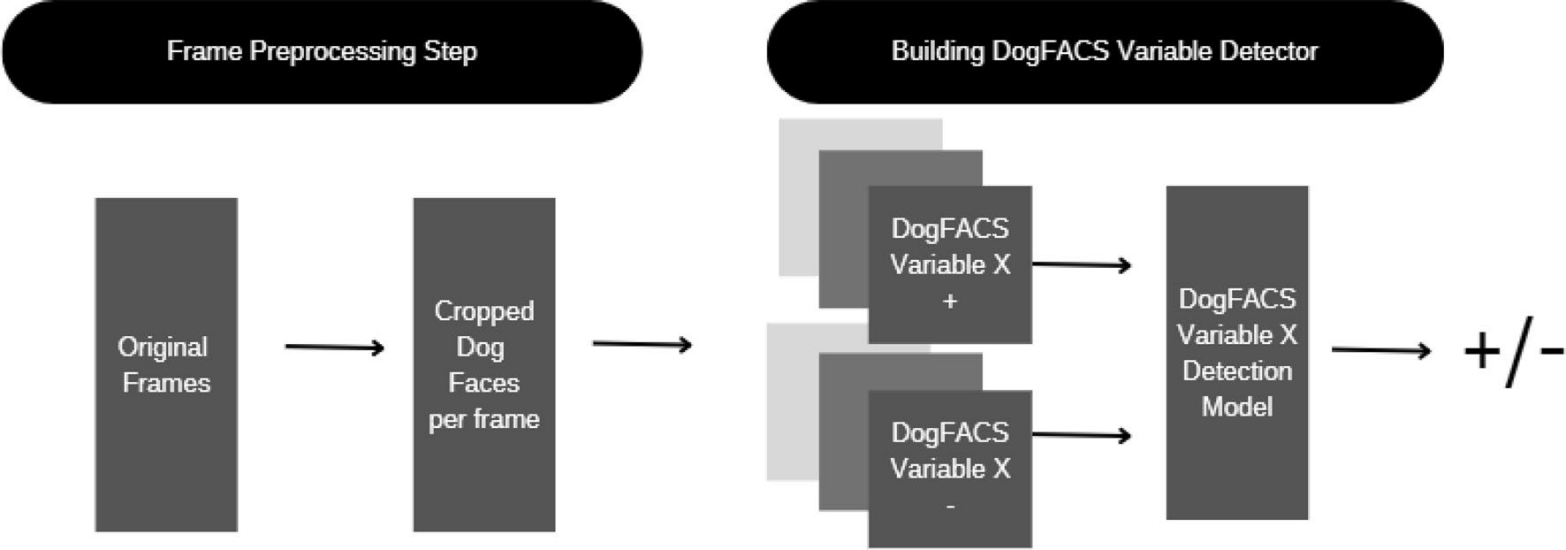
Frame to DogFACS variable detector pipeline description.

The full pipeline is described in the following diagram see Fig. 9. It includes the following steps:

- *Cropping* dog faces out of the frames using the method described above.
- *Building DogFACS variable datasets* Using manual DogFACS coding of Bremhorst et al.^22^, for every DogFACS variable, we created two folders with positive and negative examples (dog face either expressing or not expressing this DogFACS variable). For the positive samples (variable present), we selected the images of all frames manually coded with this variable. For the negative samples, we selected frames in videos not having the variable marked on their coding until the first appearance of that variable (or until the end of the video if not present). The datasets were then balanced, leaving an equal number of images for positive and negative examples for each variable. Table 4 shows the size of the datasets for all DogFACS variables for which detectors were obtained.

#### From DogFACS variables to classification of emotional states

We used transfer learning based on a pre-trained ResNet50 network architecture initialized with Imagenet weights. We replaced its top layer with an average pool layer, a 20 percentage drop out layer and a two classes classifier layer. The model was trained during 20 epochs using Adam optimizer with learning rate of 0.0001. The model achieving maximal accuracy on the validation dataset was selected as the best model. During the first 10 epochs, the weights of all layers were fine-tuned. During first 10 epochs, weight of all layers were fine-tuned. During the the remaining epochs, ResNet50 weights were frozen and only weights of new top layers were updated. For non-orientation related variables (‘Ears Flattener’, ’Lips Part’, ‘Ears Adductor’, ‘Ears Forward’ and ‘Nose Lick’) we applied an augmentation technique based on random image horizontal flip and rotation of up to 20°. As input for the encoder we used an input table, where each row represents the presence (1)/absence (0) of each of the 11 DogFACS variables on each video. The target of the encoder is a table containing the condition (negative(0)/positive(1)) of each video.

### Deep approach

Until recently convolutional neural networks (CNNs) were considered state of the art in computer vision tasks. Recently the Vision Transformer (ViT)^57^ architecture emerged as an alternative^73^. The DINO method for training has only been introduced in 2021 as a self-distillation learning frame. Training several DNN backbones (ResNet50, vit-small, vit-base etc) in this configuration it was shown that a ViT backbone trained with DINO approach outperforms previous classification results on ImageNet standard dataset^74^.

**Figure 10:**
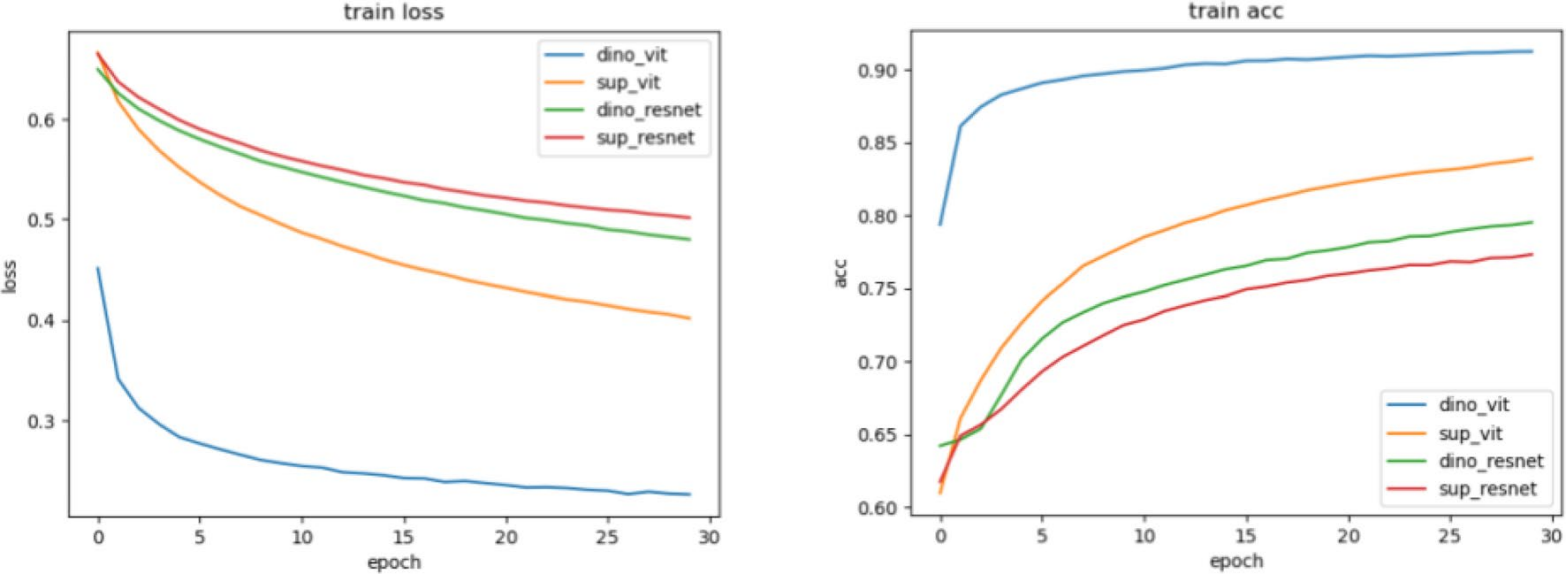
Loss and accuracy curves for each model. The loss and accuracy graphs on the train set for each trained model. The DINO-ViT based model performs better than models based on other backbones.

We used ResNet50 architecture for supervised and DINO-trained backbones; ViT-S/16 trained in a supervised manner and ViT-S/8 trained with DINO. We use pretrained ViT weights from the Timm Library^75^. We train all the four models for 30 epochs using Adam optimizer^76^ with betas = (0, 0.999) and learning rates: $10^{-4}$ for ResNet backbones and $5\cdot 10^{-6}$ for ViT backbone. The loss curves of the trained models are presented on Fig. 10.

#### Map visualization

We opt for the Eigen-CAM method^59^ to visualize the principal components of the final activations for each model. It has been shown that Eigen-CAM provides more easily interpretable results with less computation compared to other CAM methods such as the popular Grad-CAM^77^. Moreover, unlike other visualization methods such as Grad-CAM^59^ and Grad-CAM++^78^, Eigen-CAM is a class-independent tool. This property enables Eigen-CAM to visualize learned patterns even when the model prediction is wrong, as opposed to older CAM methods that produce irrelevant maps when their prediction is incorrect. This property of Eigen-CAM enables interpreting reasons for prediction failure. It is more consistent and class discriminative compared to other state of the art visualization methods. In addition, EigenCAM is not model-specific—it can be used for both ViTs and CNNs without changing layers.

## Data Availability

The dataset used in this paper is available upon request from the corresponding author.
